# Guided Conditional Diffusion Classifier (ConDiff) for Enhanced Prediction of Infection in Diabetic Foot Ulcers

**DOI:** 10.1109/OJEMB.2024.3453060

**Published:** 2024-09-02

**Authors:** Palawat Busaranuvong, Emmanuel Agu, Deepak Kumar, Shefalika Gautam, Reza Saadati Fard, Bengisu Tulu, Diane Strong

**Affiliations:** Data Science DepartmentWorcester Polytechnic Institute 8718 Worcester MA 01609 USA; Computer Science DepartmentWorcester Polytechnic Institute 8718 Worcester MA 01609 USA; Business SchoolWorcester Polytechnic Institute 8718 Worcester MA 01609 USA

**Keywords:** Diabetic foot ulcers, diffusion models, distance-based image classification, generative models, wound infection

## Abstract

*Goal:* To accurately detect infections in Diabetic Foot Ulcers (DFUs) using photographs taken at the Point of Care (POC). Achieving high performance is critical for preventing complications and amputations, as well as minimizing unnecessary emergency department visits and referrals. *Methods:* This paper proposes the Guided Conditional Diffusion Classifier (ConDiff). This novel deep-learning framework combines guided image synthesis with a denoising diffusion model and distance-based classification. The process involves (1) generating guided conditional synthetic images by injecting Gaussian noise to a guide (input) image, followed by denoising the noise-perturbed image through a reverse diffusion process, conditioned on infection status and (2) classifying infections based on the minimum Euclidean distance between synthesized images and the original guide image in embedding space. *Results:* ConDiff demonstrated superior performance with an average accuracy of 81% that outperformed state-of-the-art (SOTA) models by at least 3%. It also achieved the highest sensitivity of 85.4%, which is crucial in clinical domains while significantly improving specificity to 74.4%, surpassing the best SOTA model. *Conclusions:* ConDiff not only improves the diagnosis of DFU infections but also pioneers the use of generative discriminative models for detailed medical image analysis, offering a promising approach for improving patient outcomes.

## I. Introduction

Chronic wounds, affecting over 6.5 million people or approximately 2% of the U.S. population, represent a significant health issue with healthcare expenses exceeding $25 billion each year [1], [2]. Diabetic Foot Ulcers (DFUs), a prevalent subtype of chronic wounds, pose substantial risks for diabetic patients. Often located on the soles of the feet, DFUs are highly susceptible to infection, with 40% to 80% of cases becoming infected [3].

*The problem:* Accurate diagnosis of infections in DFUs involves analyzing the bacteriology of the wound and reviewing patient records, including clinical history, physical health assessments, and blood tests. However, as clinicians do not always have access to this comprehensive wound information, they often rely on visual inspection to identify signs of infection in DFUs. Visual indicators of infection include increased redness around the ulcer and colored purulent discharge. In addition, at the point of care (for example, patient homes or trauma sites), routine wound assessment and infection detection are frequently performed by caregivers who are not wound experts, especially in low-resource settings and developing countries. When such caregivers suspect an infection, they err on the side of caution by recommending that patients visit a clinic or emergency services where a wound expert checks to determine if the wound is infected. For wounds that are actually infected, the referral process can delay treatment for infected wounds, increasing the likelihood of complications, including amputations, surgery that costs between $20,000 and $60,000 per patient [11], [12] and has associated lifetime rehabilitation costs of $509,272 [13]. Unfortunately, approximately half of the patients die within five years after a lower extremity amputation [14]. If the wound is not infected, the referral process wastes patient and provider time and incurs unnecessary expenses such as transportation and emergency department costs [15], [16].

This paper proposes an automated deep-learning method for effective monitoring and early detection of infections in DFUs from images at the Point of Care (POC).

*Prior work:* Recently, machine learning methods have achieved impressive performance in various medical image analysis and wound assessment tasks, including the works by Liu et al. [17] to score the healing progress of chronic wounds from photographs based on evidence-based rubrics, such as the Photographic Wound Assessment Tool (PWAT). Furthermore, State-of-the-Art (SOTA) machine learning techniques have been proposed to detect infections from the visual appearance of wounds on photographs [6], [7], [9], [10] without the need for direct wound tests, medical notes, or extensive clinical examinations. Goyal et al. [6] introduced the CNN-Ensemble model, which extracts bottleneck features from CNN architectures that are then classified using an SVM classifier. CNN-Ensemble achieved 72.7% accuracy for binary infection classification of wound images in the DFU infection dataset.

**Fig. 1.**
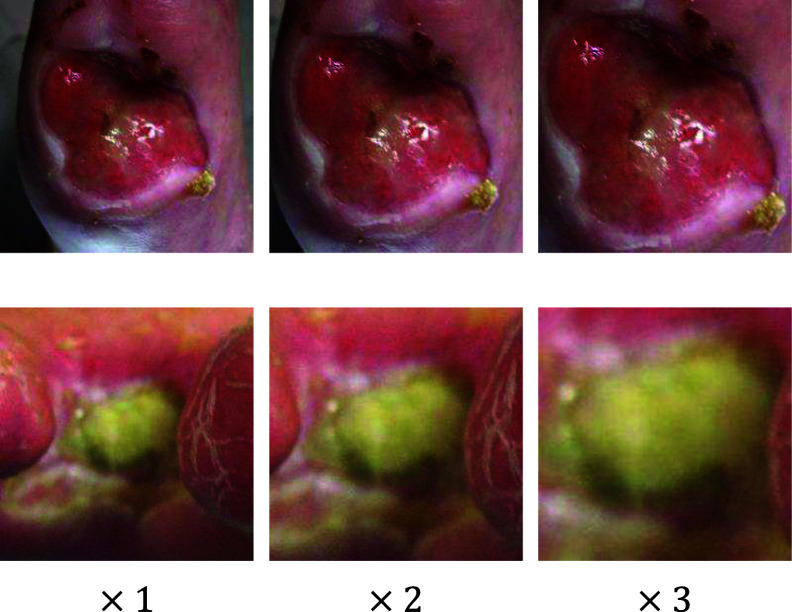
Natural data augmentation of an original image with three different magnifications.

In a subsequent study, Liu et al. [8] reported an impressive accuracy of 99% for wound infection classification by adapting the EfficientNet model [18], along with data augmentation techniques. However, their high accuracy was due in part to data leakage issues between training and testing data sets. Specifically, the original DFU infection dataset from Goyal et al. [6] included each wound image in three naturally augmented forms with varying magnifications (see Fig. 1). Liu et al. [8] randomly split these augmented images between training and testing sets on a sample-wise basis, which resulted in significant data leakage since the testing set included images that closely resembled augmented versions of the training images.

*Challenges:* Detecting infection in DFU images using deep learning faces several obstacles. First, the distinction between infected and uninfected wounds is subtle, with high inter-class similarity and intra-class variation [6], complicating accurate classification. Second, wound image datasets often suffer from inconsistent imaging conditions, such as variations in camera distance, orientation, and lighting [6].

**Fig. 2.**
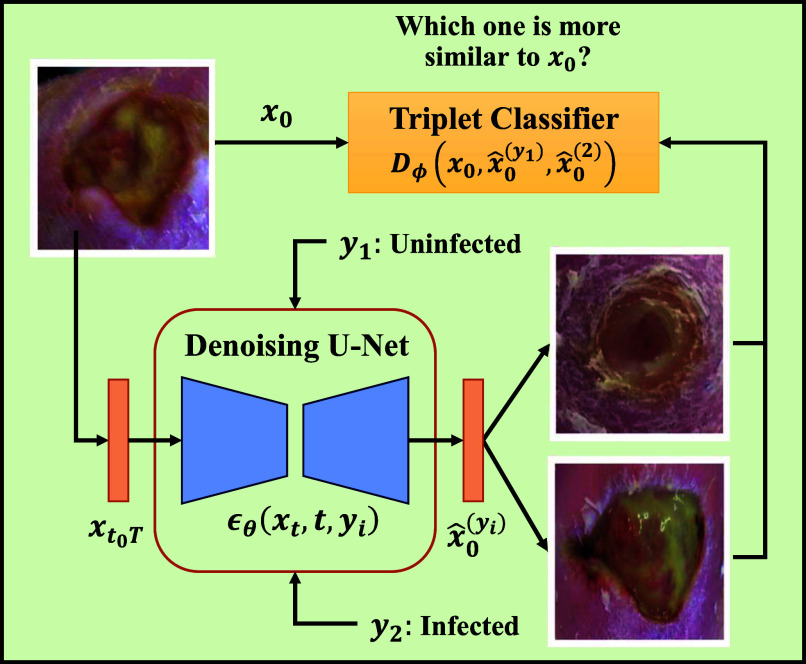
Inference in the ConDiff Classier Framework. Input $x_{0}$ is perturbed by noise of strength $t_{0}$. The perturbed input $ x_{t_{0}T}$ is denoised through a reverse diffusion process to synthesize image $\hat{x}_{0}^{(y_{i})}$ conditioned on label $y_{i}$. Infection classification is based on the minimum $L_{2}$ distance between $x_{0}$ and $\hat{x}_{0}^{(y_{i})}$ in embedding space.

*Our approach:* This paper presents the Guided Conditional Diffusion Classifier (ConDiff), a novel generative discriminative approach for wound infection classification (see Fig. 2). ConDiff leverages conditional guide image editing with a generative diffusion model [19], [20] by perturbing an input image with a specific amount of Gaussian noise, and generating new images by using a reverse diffusion process to gradually remove noise from the noise-perturbed input image. The ConDiff diffusion process is conditioned on the state of the wound (no infection ($y_{1}$) or infection ($y_{2}$)), creating synthetic images reflective of these states. One key importance is the ability of ConDiff to discern and learn similarities between the representations of the conditionally generated images $\hat{x}^{y}_{0}$ and the original wound image $x_{0}$ through a distance-based classifier $L_{2}$ in the embedding space. The condition that yields the synthetic image that is most similar to the original is selected as the predictive label. This work utilizes the DFU infection dataset provided by Goyal et al. [6] (see Table I). However, to eliminate data leakage between training and test sets, we have refined our dataset creation and splitting strategy. Using subject-wise splitting, only the second magnified naturally augmented image (refer to Fig. 1) is utilized for each subject.

**TABLE I:** Summary of Prior Work on Wound Infection Classification Using Deep Learning

**Specific ML problem**	**Related Work**	**Summary of Approach**	**No. of Target Classes**	**Dataset**	**Results**
Wound segmentation and Infection Classification	Wang et al. 2015 [4]	CNN-based: ConvNet + SVM	2 classes (infection and no infection)	NYU wound Database	Accuracy: 95.6% PPV: 40% Sensitivity: 31%
Classification of 7 tissue types including infection	Nejati et al. 2018 [5]	CNN-based: AlexNet + PCA + SVM		**Private data (data statistics is unknown)**	**Accuracy 95.6% (Only reported accuracy)**
DFU infection classification	Goyal et al. 2020 [6]	CNN-based: Ensemble CNN	2 classes (infection and no infection)	*Part B DFU 2020 dataset (We also used this dataset)*	Accuracy: 72.7% PPV: 73.5% Sensitivity: 70.9%
	Al-Garaawi et al. 2022 [7]	CNN-based: DFU-RGB-TEX-Net			Accuracy: 74.2% PPV: 74.1% Sensitivity: 75.1%
	Liu et al. 2022 [8]	CNN-based: augmentations + EfficientNet			**Data leakage** when splitting & performing augmentations
DFU wound ischemia and infection classification	Yap et al. 2021 [9]	CNN-based: VGG, ResNet, InceptionV3, DenseNet, EfficientNet	4 classes (both infection and ischemia, infection, ischemia, none)	DFUC2021 dataset	EfficientNet B0 performance: F1, PPV, SEN = 55%, 57%, 62%
	Galdran et al. 2021 [10]	ViT-based: ViT, DeiT, BiT			BiT performance: F1, PPV, SEN = 61%, 66%, 61%

*Main contributions:*

• We propose the Guided Conditional Diffusion Classifier (ConDiff), an end-to-end framework that uniquely integrates a guided diffusion and a distance-based classifier for classifying infected wound images. To the best of our knowledge, ConDiff is the first generative discriminative method to analyze fine-grained wound images.
• In evaluations using 5-fold cross-validation on the test DFU dataset (148 infected, 103 uninfected), ConDiff consistently outperforms SOTA models, achieving up to the highest sensitivity of 85.4% and demonstrating superior generalization through low standard deviations across different test folds.
• Heatmaps generated by Score-CAM [21] are applied to visually illustrate that ConDiff focuses on the correct wound regions when classifying wound infection status.

## II. Methodology

We now focus on the ConDiff framework, its components, theoretical bases, and practical application.

### A. Denoising Diffusion Model

Denoising Diffusion Probabilistic Model (DDPM) [22] is a generative model that leverages diffusion processes to generate synthetic data. DDPM has two main stages: 1) a forward process and 2) a reverse process. The forward process $q(x_{1:T}|x_{0})$ incrementally adds Gaussian noise to an initial image $x_{0}$ in $T$ steps, ultimately transforming it into a Gaussian distribution $p(z)$. In the reverse process $p_\theta (x_{0:T})$, the model learns to remove this noise iteratively to reconstruct or generate data samples, accomplished using a neural network $\epsilon _\theta$ trained to predict the noise added to the noisy image $x_{t}$ at each step $t$.

#### 1) Conditional Image Generation With Diffusion Models

The conditioning variable $y$ is considered as an additional input to the denoising network represented as $\epsilon _\theta (x_{t},t,y)$. To synthesize high-quality images, the *Classifier-Free Guidance (CFG)* [23] introduced the Guidance Scale $\omega$ to control how much the generated data is influenced by a condition $y$ as illustrated in (1). A higher $\omega$ means more influence from $y$. See the Supplementary Materials for derivation.

\begin{equation*}
\tilde{\epsilon }_\theta (x_{t},t,y) = (1-\omega)\varepsilon _{\theta} (x_{t},t) + \omega \epsilon _\theta (x_{t},t,y) \tag{1}
\end{equation*}

#### 2) Learning Objective

In this work, the conditional denoising model $\epsilon _\theta (x_{t},t,y)$ is modeled with a U-Net architecture and is optimized by minimizing the mean square error (MSE) between the actual and predicted noise (2).

\begin{equation*}
L_{DM}(\theta) = \mathbb {E}_{x_{0},t,y,\epsilon \sim \mathcal {N}\,(0,I)}\left[ {\Vert \epsilon -\epsilon _\theta (x_{t},t,y)\Vert }^{2}_{2} \right] \tag{2}
\end{equation*}

### B. Guided Conditional Diffusion Classifier (ConDiff)

To complete our ConDiff model, two additional components are now introduced: 1) guided image synthesis and 2) triplet loss for learning similarity.

#### 1) Guided Image Synthesis

We aim to generate conditional images that are guided by the original images. Specifically, we seek to synthesize wound images that closely resemble the input image, while also being distinct enough to differentiate between infection and non-infection conditions. We employ a strategy from image synthesis with Stochastic Differential Equations (SDEs) [20], introducing a certain amount of Gaussian noise to the guide image through a forward diffusion process (3). Next, we synthesize conditional images using classifier-free guidance (Section II-A1). The noise strength $t_{0} \in (0,1]$ indicates the level of noise added to the original image $x_{0} \sim q(x)$:

\begin{equation*}
x_{t_{0}T} = x_{0} + \sigma (t_{0})z, \text{ where } z \sim \mathcal {N}\,(0,I) \tag{3}
\end{equation*}

**Fig. 3.**
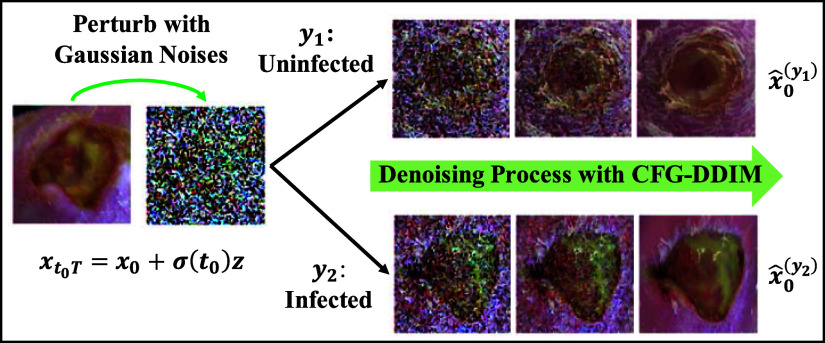
Synthesizing conditional DFU images using ConDiff. A guide image $x_{0}$ is perturbed with Gaussian noises that are then removed progressively using a CFG-DDIM sampling technique, conditioned on the infection status. This process gradually projects $x_{0}$ to guided synthetic images of conditions: $\hat{x}_{0}^{(y_{1})}$ and $\hat{x}_{0}^{(y_{2})}$.

Here, $\sigma (t_{0})$ is a scalar function determining the noise magnitude, and $T$ is the total number of forward diffusion steps. Subsequently, to accelerate the sampling process, the Denoising Diffusion Implicit Model (DDIM) [24] is combined with the CFG. The CFG-DDIM sampling process is illustrated in Fig. 3 and its algorithm is described in the supplementary materials.The CFG-DDIM sampling process and its algorithm are described in the supplemental materials.

#### 2) Learning Similarity With Triplet Loss

Our next goal is to identify which synthesized image most closely resembles the guide image $x_{0}$. This is achieved using the triplet loss function (4) [25], which minimizes the distance between an anchor image $x^{(a)}$ and a positive image $x^{(p)}$ (same identity), while maximizing the distance between the anchor and a negative image $x^{(n)}$ (different identity). The embedding network $f_\phi (x)$ maps the images to a $d$-dimensional Euclidean space for similarity comparison.

\begin{align*}
 L_{triplet} =& \mathbb {E}\bigg [ \left(\Vert f_\phi (x^{(a)}) - f_\phi (x^{(p)})\Vert ^{2}_{2} \right. \\
& \quad\left. - \Vert f_\phi (x^{(a)}) - f_\phi (x^{(n)})\Vert ^{2}_{2} + \alpha \right)_+ \bigg ] \tag{4}
\end{align*}

With a margin $\alpha$ set to 1, this function enforces the desired separation between similar and dissimilar pairs. Our classifier $D_\phi$ then identifies the closest synthesized image to the guide image $x_{0}$ by comparing their $L_{2}$ distance in embedding space (5).

\begin{equation*}
D_\phi (x_{0},\hat{x}_{0}^{(y_{1})},\hat{x}_{0}^{(y_{2})}) = \arg \min _{y_{i}} \left\lbrace L_{2} (f_\phi (x_{0}),f_\phi (\hat{x}_{0}^{(y_{i})})) \right\rbrace \tag{5}
\end{equation*}

## III. Experiments and Results

### A. Diabetic Foot Ulcer (DFU) Dataset

The DFU Infection Dataset curated by Goyal et al. [6], comprises DFU images collected at the Lancashire Teaching Hospital with the permission of the U.K. National Health Service (NHS). These DFU images were labeled by two DFU specialists (consultant physicians) based on visual inspection, independent of medical notes or clinical tests. The dataset consists of 2,946 augmented patches with infection and 2,946 augmented patches without infection. Natural augmentation was performed with varying magnification as illustrated in Fig. 1. All patches have dimensions of $224 \times 224 \times 3$ pixels.

*Data Preprocessing:* The training, validation and test sets were created using 70%, 15% and 15% of the dataset respectively. To prevent data leakage, subject-wise splitting was utilized, where all images for each case could only belong to one class. As a further measure to prevent data leakage, as shown in Fig. 1, only augmented patches with a $\times 2$ magnification level were considered. The model achieving the highest accuracy on the validation set (optimal parameter values) was selected for final evaluation on the test (unseen) dataset. Table II

**TABLE II:** Refined DFU Dataset Statistics

Processed Data	Category	# of Patches
Train Data (70%)	Infection	687
	No Infection	480
Validation Data (15%)	Infection	147
	No Infection	102
Test Data (15%)	Infection	148
	No Infection	103

shows the dataset statistics after pre-processing.

### B. Experimental Setup

#### 1) Implementation Details

The ConDiff classifier was trained in two distinct stages on an NVIDIA A100 GPU.

*Training Stage 1 - Fine-Tuning the Diffusion Model:* The diffusion model $\epsilon _\theta (x_{t},t,y)$ was fine-tuned using the objective function defined in (2). After training, the ConDiff generator synthesized conditional DFU images with hyperparameters: guidance scale $\omega =0.75$, noise strength $t_{0}=0.8$, and number of sampling steps $T=30$. These synthetic images formed the dataset $D_{s}$, used in the second training stage.

*Training Stage 2 - Training the Embedding Network $f_\phi$:* The embedding model $f_\phi$, based on the EfficientNet-B0 architecture, was trained using both real dataset $D_{r}$ and synthetic dataset $D_{s}$. For each iteration, a batch of triplets $(x^{(a)}, x^{(p)}, x^{(n)})$ was sampled from $D_{r}$, with $(x^{(p)}, x^{(n)})$ being sampled from $D_{s}$ with probability $p_{gen}=0.2$. The model parameters $\phi$ were optimized to minimize the triplet loss. Please see the Supplementary Materials for more details on the 2-step training process.

#### 2) SOTA Baseline Models

Recent deep learning image classification architectures including CNN and ViT-based models were considered as baselines. Due to the small size of our dataset, the base or tiny version of each model was selected for evaluation.

*CNN-based models:* ResNet [26] and Inception-V3 [27] were selected as baseline models because Goyal et al. [6] employed them as backbones in the ensemble CNN model for DFU infection classification. DenseNet [28] was selected as Yap et al. [9] found that it achieved the best macro-F1 score in 4-class DFU image classification. EfficientNet [18] was selected as it was the most effective CNN-based model in analyzing wound infections [9], [10].

*ViT-based models:* ViT [29] and DeiT [30] were explored for DFU ischemia & infection classification by Galdran et al. [10], achieving a macro-F1 score comparable to the best CNN-based model (EfficientNet). SwinV2 [31] and EfficientFormer [32] were selected as baselines for DFU infection classification because even have not previously been explored for infection classification, they are recent architectures that outperformed previous ViT-based & CNN-based models on ImageNet classification.

### C. Performance Comparison With SOTA Baselines

Table III shows that ConDiff achieves the highest accuracy of up to 81%, outperforming SOTA baselines by at least 3% in a 5-fold cross-validation on the test set. Moreover, its sensitivity of 85.4% is the highest among all baseline models, slightly surpassing the EfficientFormer model by 1% (85.4% vs. 84.1%). High sensitivity is crucial for detecting infected wounds early, significantly reducing severe consequences such as amputations and other complications arising from delayed treatment.

**TABLE III:** Quantitative Comparison of ConDiff and SOTA Baseline Models on DFU Infection Test Images

Model		Accuracy	F1-score	Sensitivity	Specificity	PPV
Convolutional Neural Networks	ResNet-18	0.765 (0.015)	0.799 (0.015)	0.796 (0.032)	0.720 (0.041)	0.803 (0.019)
	DenseNet-121	0.750 (0.033)	0.782 (0.029)	0.759 (0.034)	0.737 (0.044)	0.806 (0.031)
	Inception-V3	0.745 (0.015)	0.780 (0.021)	0.769 (0.052)	0.712 (0.054)	0.794 (0.021)
	EfficientNet-B0	0.766 (0.035)	0.793 (0.038)	0.766 (0.072)	**0.766** (0.066)	0.827 (0.036)
Vision Transformers	ViT-Small	0.765 (0.024)	0.798 (0.035)	0.803 (0.087)	0.710 (0.090)	0.803 (0.034)
	DeiT-Tiny	0.769 (0.022)	0.800 (0.025)	0.789 (0.052)	0.739 (0.056)	0.814 (0.025)
	SwinV2-Tiny	0.774 (0.022)	0.811 (0.021)	0.827 (0.040)	0.698 (0.040)	0.797 (0.020)
	EfficientFormer-L1	0.780 (0.023)	0.818 (0.015)	0.841 (0.032)	0.692 (0.080)	0.799 (0.036)
Diffusion	**ConDiff (ours)**	**0.810** (0.020)	**0.841** (0.017)	**0.854** (0.017)	0.747 (0.027)	**0.828** (0.018)

Bold Values Indicate the Highest Scores and Underlined Values Represent the Second-Highest.

In addition to its impressive sensitivity, ConDiff also achieves an excellent specificity of 74.7%, which is substantially higher than EfficientFormer's 69.2%. Higher specificity implies that ConDiff can reduce incorrect referrals, thus reducing the workload of wound experts in clinics and reducing patient anxiety and frustration.

The combination of high sensitivity and specificity makes ConDiff an ideal model for the early detection of infected wounds at the POC. While its sensitivity score is only marginally better than EfficientFormer's, the significant improvement in specificity ensures not only better patient outcomes but also reduces the susceptibility to alarm fatigue from false positives and allows for more efficient utilization of clinical resources.

**Fig. 4.**
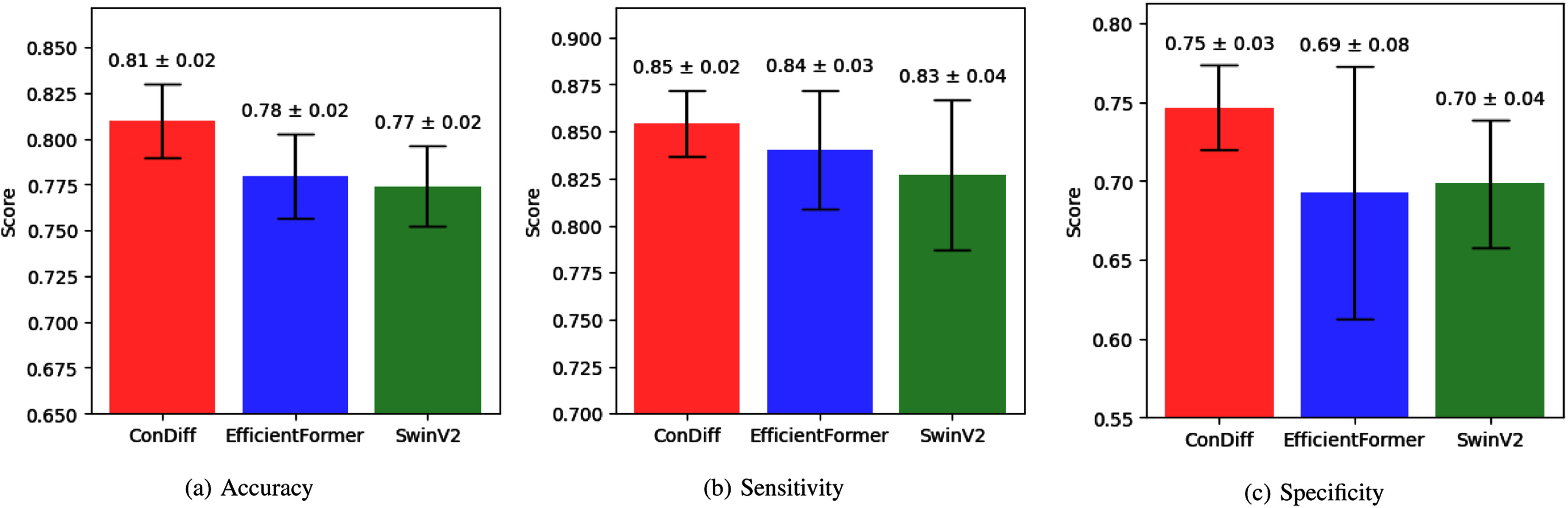
Bar chart comparing (a) accuracy, (b) sensitivity and (c) specificity of ConDiff (shown in red) and the top-2 Vision-Transformer-based models (EfficientFormer in blue, and SwinV2 in green). The scores are averaged over a 5-fold cross-validation, with error bars indicating the standard deviation.

Beyond improved evaluation scores, ConDiff exhibits lower standard deviations and less variation in performance across different folds compared to other models, indicating robust generalization to unseen wound images (see the bar chart comparison in Fig. 4). This robustness is particularly crucial in a clinical setting, where wounds of various appearances are commonly encountered. For instance, the standard deviation of the sensitivity score for ConDiff is 1.7%, compared to 3.2% for EfficientFormer, and the standard deviation of the specificity score for ConDiff is 2.7%, compared to 8.0% for EfficientFormer.

Another notable observation from Table III is that ViT-based models achieved slightly better performance compared to CNN-based models. This improvement can be attributed to the attention mechanisms used by ViT-based models, which effectively capture global dependencies between all input data elements. In contrast, CNN-based models apply uniform filters across the entire image, focusing primarily on local content. This local focus makes CNNs less suited for capturing high inter-class similarity and intra-class variation, which are essential for accurate wound detection and classification.

**Fig. 5.**
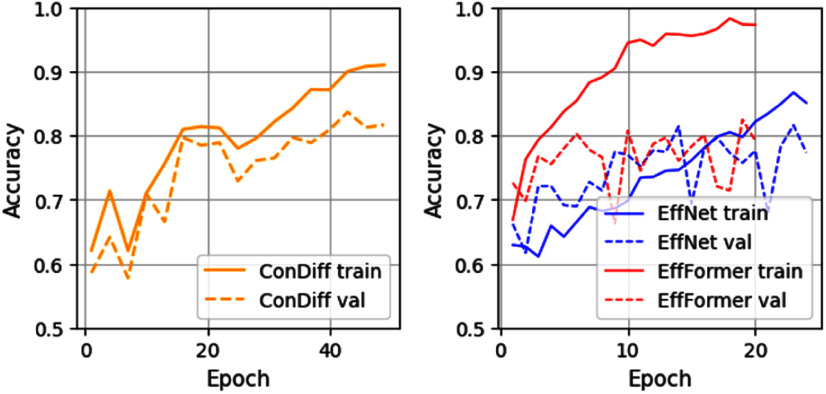
The learning accuracy trajectories of the ConDiff classifier and best-performing CNN-based (EfficientNet-B0) and ViT-based (EfficientFormer-L1) models, on the train and validation sets.

However, ViT-based models tend to overfit on our relatively small training dataset, as illustrated in Fig. 5 (Right). In contrast, the EfficientNet-B0 model is less susceptible to overfitting than the EfficientFormer-L1 model. This led us to select EfficientNet-B0 as the embedding network $f_\phi$ in the ConDiff classifier. Consequently, as shown in Fig. 5 (Left), ConDiff effectively mitigates overfitting. This is attributed to the use of the triplet loss function, which enables it to learn to distinguish between similar and dissimilar images based on Euclidean distances in the embedding space.

#### 1) Explaining Image Similarity in Embedding Space With Score-CAM

**Fig. 6.**
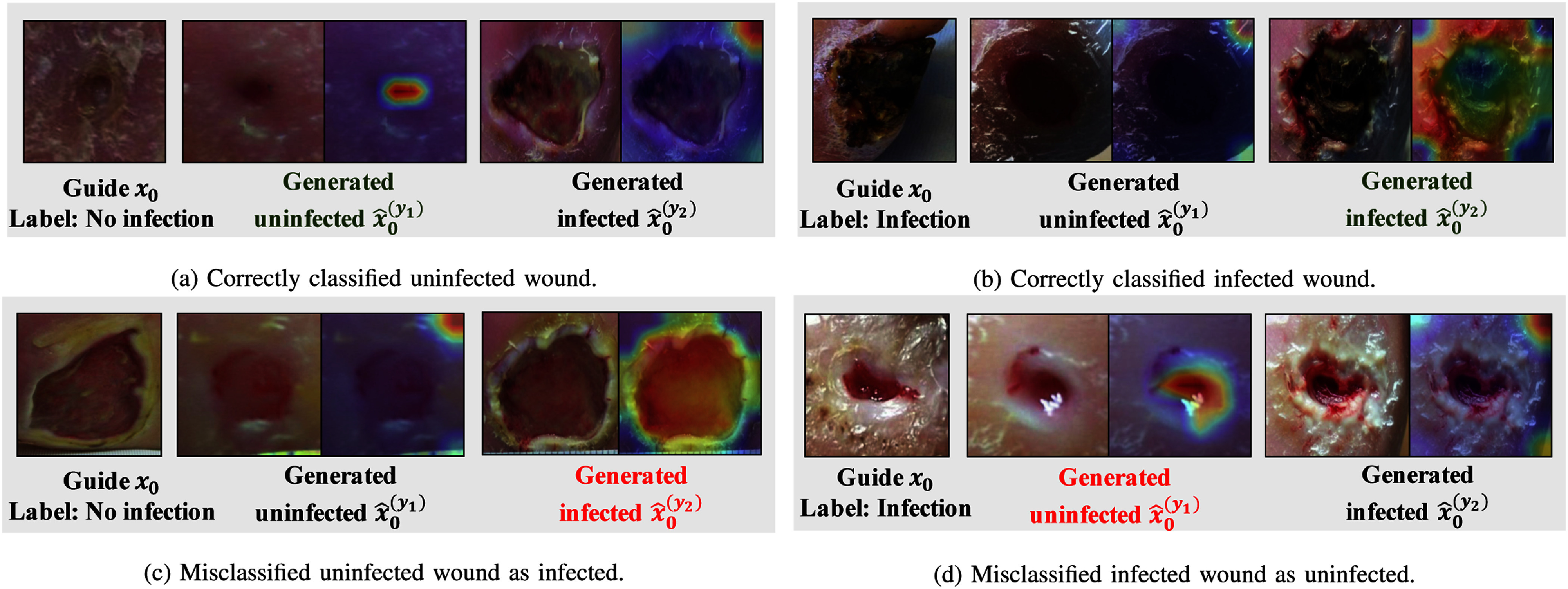
Visualization of ConDiff predictions with corresponding Score-CAM images computed from the ConDiff's embedding model $f_\phi$. Each sub-figure shows an example with the guide image ($x_{0}$), conditional synthesized images representing uninfected $\hat{x}_{0}^{(y_{1})}$ and infected $\hat{x}_{0}^{(y_{2})}$ states, and their respective Score-CAM overlays indicate regions with similar features to $x_{0}$.

Fig. 6 presents Score-CAM [21] visualizations that elucidate similarities the embedding model perceives between conditional synthesized images and their corresponding guide images $x_{0}$. The detailed description of the Score-CAM algorithm is illustrated in the Supplementary Materials. Areas highlighted in red on the Score-CAM heatmaps shown in Fig. 6 denote regions that the ConDiff classifier identified as having a high degree of similarity to $x_{0}$. For instance, Fig. 6(a) illustrates that the classifier recognizes similar features in a synthesized uninfected image and the guide image, as indicated by the presence of a red spot in the heatmap. Conversely, the heatmap corresponding to the generated image conditioned on DFU infection does not reveal substantial similarity, except for a marginal overlap in the background at the top-right corner. Similarly, Fig. 6(b) depicts an accurate detection of infection, where the embedding model $f_{\phi }$ concentrates on the necrotic tissue evident in both $x_{0}$ and $\hat{x}_{0}^{(y_{2})}$. However, the classifier is not infallible. Examples of misclassifications are demonstrated in Fig. 6(c) and (d), where the embedding model incorrectly assesses a synthesized image conditioned on a different class as being more similar to the guide image, an error attributable to the high inter-class similarity in embedding space.

#### 2) Exploring Mis-Classifications

**Fig. 7.**
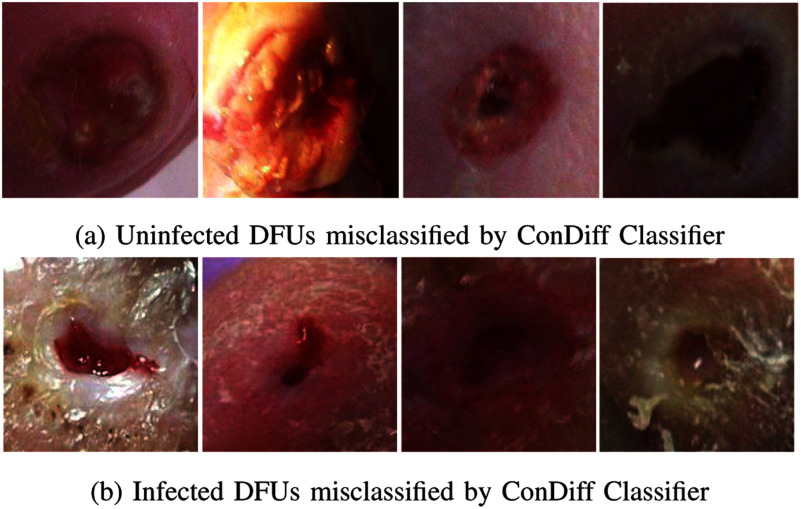
Examples of incorrectly classified DFU images for infection by our ConDiff Classifier.

Fig. 7(a) and (b) show misclassified cases. The uninfected DFUs in Fig. 7(a) resemble infected wounds exhibiting characteristics such as a large reddish area or darkening of the wound, possibly caused by poor lighting conditions. The misclassified examples in Fig. 7(b) are due to the small size of the wounds and ambiguous features, such as a somewhat dry appearance, which confuses the infection classifier.

#### 3) Effects of Noise Strengths $t_{0}$ on Infection Classification

This experiment involves varying noise strengths $t_{0}$ to the input images. The guidance scale $\omega$ was fixed at 7.5.

**Fig. 8.**
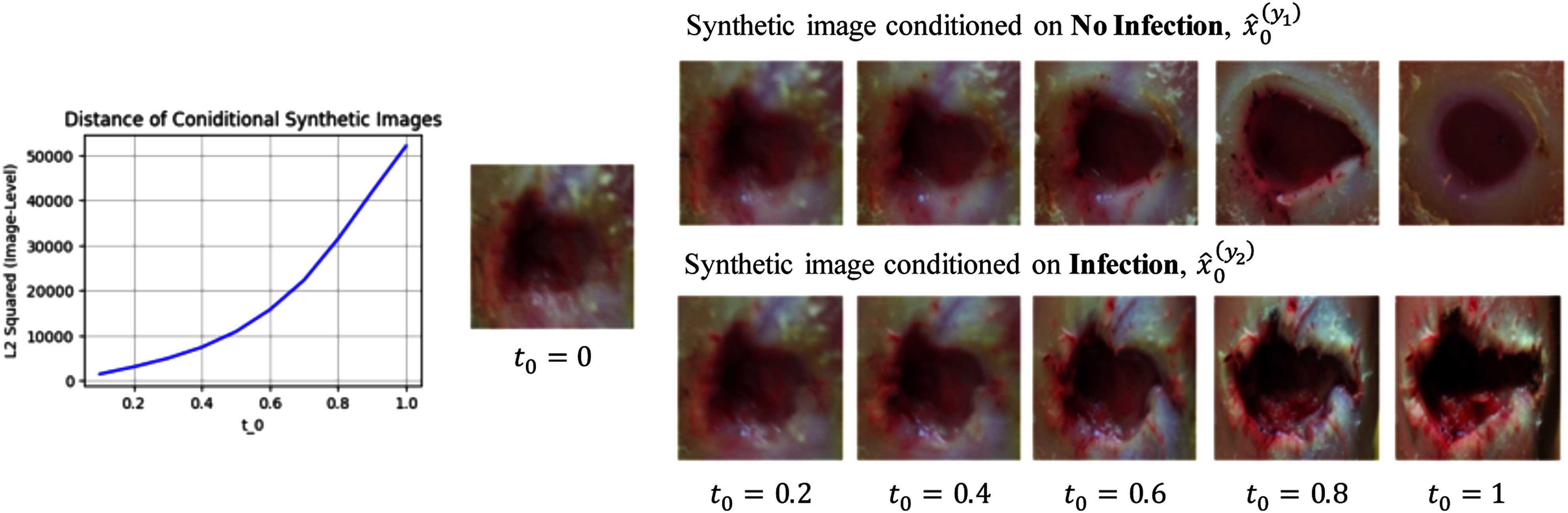
(Left) $L_{2}$ norm squared between $\hat{x}_{0}^{(y_{1})}$ and $\hat{x}_{0}^{(y_{2})}$ plot with respect to initial noise perturbation steps $t_{0}$, (Right) illustration of conditional synthesized images of ConDiff + CFG-DDIM sampling: $\omega =7.5$ with various $t_{0}$ initialization. As $t_{0}$ increases, the difference in image level between conditional generated images increases.

Table IV highlights the significant role of perturbed noise in image synthesis. As the noise strength $t_{0}$ increases, the guided synthesized images corresponding to different labels exhibit greater divergence, facilitating more straightforward predictions by our distance-based classifier $D_\phi$. However, when $t_{0}$ is increased beyond certain thresholds, the distances between input images and their respective guided synthesized counterparts become excessively large for both labels. This increase in distance diminishes the distinguishability of $D_\phi$. Classifications with $t_{0}$ values ranging from 0.1 to 0.4 were excluded from our analysis, as depicted in Fig. 8 (Left), where it is shown that the average difference in the $L_{2}$ norm squared between the two guided synthetic images $\hat{x}_{0}^{(y_{1})}$ and $\hat{x}_{0}^{(y_{2})}$ is relatively small. Fig. 8 (Right) presents examples of conditional synthetic images generated by ConDiff + CFG-DDIM sampling across different $t_{0}$ values.

**TABLE IV:** Quantitative Comparison of the Result of DFU Infection Classification on the Test Data by ConDiff Classifier With Different Strengths $t_{0}$ of Perturbed Noise

$t_{0}$	Acc	F1	SEN	SPEC	PPV
0.5	0.721	0.761	0.757	0.670	0.767
0.6	0.721	0.757	0.736	0.699	0.779
0.7	0.773	0.800	0.770	0.777	0.832
0.8	**0.833**	**0.858**	**0.858**	0.796	0.858
0.9	0.809	0.829	0.791	**0.835**	**0.873**

The bold values indicate the highest scores.

## IV. Discussion

*Summary of Findings:* **The ConDiff classifier outperforms other deep learning models in detecting infections in DFU images** by minimizing triplet loss instead of binary cross-entropy loss, enabling it to effectively match input images with the most similar conditionally synthesized images in embedding space.

*Overfitting Mitigation:* ConDiff's training strategy in *Training Stage 2*, involving triplet loss, not only enhances its performance but also reduces overfitting by learning to discern between infected and uninfected wounds in the dataset.

*Score-CAM enhances ConDiff's Interpretability:* As shown in Fig. 6, ConDiff focuses on wound features critical for accurately predicting infection status in its decision-making.

*Clinical Significance:* The ConDiff framework not only outperforms other SOTA baselines in sensitivity but also achieves high specificity, allowing wound experts to focus on more severe cases. This advancement ensures accurate and efficient wound infection detection, ultimately improving patient outcomes and resource utilization. Furthermore, ConDiff exhibits lower standard deviations in different test folds, indicating better generalization and robust model performance in detecting infections from wound images.

*Model Limitation:* ConDiff's major drawback is its high computational cost during inference, taking 4-5 seconds per image on an NVIDIA A100 GPU, in contrast to less than 0.02 seconds for other models. This is due to its generative discriminative approach, which synthesizes conditional images for each input by gradually removing noise through a reverse diffusion process.

*Dataset Limitation:* The infected DFU dataset [6] lacks records or meta-data about the conditions or medical clarifications. Consequently, ConDiff's predictive framework is not to predict whether ulcers will become infected in the future. Instead, we envision an infection screening tool to assist DFU patients, who are not undergoing antibiotic treatment, in evaluating their current wound infection status at the POC.

*Future Work:* In medical contexts, model interpretability or understanding the reasons behind predictions of wound infections (e.g., signs of wound infection) is as crucial as the visual classification and interpretation themselves. Exploring multimodal data, such as incorporating thermal images or generating medical notes from Large Language Models (LLMs) could further enhance the classification capabilities of deep learning models.

## V. Conclusion

This study introduced the Guided Conditional Diffusion classifier (ConDiff), a new framework for classifying Diabetic Foot Ulcer (DFU) infections from wound images. Outperforming traditional models by at least 3%, ConDiff achieves up to 81% average accuracy. The proposed work has clinical significance as ConDiff enhances both sensitivity (85.4%) and specificity (74.4%), and also generalizes better to previously unseen wounds in different test sets, facilitating the early automated detection of infections from DFU images at the POC. Its unique approach utilizes Triplet loss instead of standard cross-entropy minimization, enhancing robustness and reducing overfitting. This is especially important in medical imaging, where datasets are often small. ConDiff employs a forward diffusion process, to add a specific amount of Gaussian noise into input images, and a reverse diffusion with classifier-free guidance to iteratively refine these images for classification based on the closest Euclidean distance in an embedding space. Its precise, real-time infection detection could play a crucial role in early DFU infection identification, reducing serious complications such as limb amputation.

## Supplementary Materials

Supplementary materials

## Author Contribution

**Palawat Busaranuvong** contributed to conceptualization, design, methodology, data analysis, and writing and editing of the manuscript. **Emmanuel Agu** was responsible for data acquisition, conceptualization, supervision, resources, manuscript review, project administration, and funding. **Deepak Kumar** provided conceptualization, supervision, and manuscript review. **Reza Saadati Fard** and **Shefalika Gautam** contributed to the methodology, review of the manuscript, and editing. **Bengisu Tulu** and **Diane Strong** managed project administration and secured funding.

## Conflict of Interest

The authors declare that they have no conflicts of interest.
