# Supplementary material for: Guided Conditional Diffusion Classifier (ConDiff) for Enhanced Prediction of Infection in Diabetic Foot Ulcers
Source: IEEE Open J Eng Med Biol. 2024 Sep 2;6:20–7. doi: 10.1109/OJEMB.2024.3453060 (PMC11573405; doi:10.1109/OJEMB.2024.3453060)
Supplement: Supplementary materials [file supp1-3453060.pdf]

Guided Conditional Diffusion Classifier (ConDiff) for Enhanced Prediction of Infection in Diabetic Foot Ulcers

Palawat Busaranuvong, Emmanuel Agu, Deepak Kumar, Shefalika Gautam, Reza Saadati Fard, Bengisu Tulu, Diane Strong

SUPPLEMENTARY MATERIALS

A. Guide Conditional Image Generation

Recent advancements have demonstrated that these models can be adapted for conditional generation, where the generated data depends on a given condition or context, such as a class label or a textual description [1]–[5]. The simplest method to implement this is to introduce the conditioning variable y as an additional input to the denoising network, represented as $\varepsilon_\theta(x_t, t, y)$. However, a limitation arises when the network does not adequately consider the conditioning variable, sometimes leading to it being overlooked entirely [6]. To address this, a "guidance scale" is introduced, enhancing the influence of the conditioning variable during sample generation.

1) Classifier-Free Diffusion Guidance: Building on the concept of guided diffusion, Ho and Salimans [3] introduced Classifier-Free Guidance (CFG) for conditional image synthesis. Utilizing Bayes' theorem, the gradient of the conditional log-likelihood, $\nabla_{x_t} \log p(x_t|y)$, can be expressed as:

$$\nabla_{x_t} \log p(x_t|y) = \nabla_{x_t} \log \frac{p(y|x_t)p(x_t)}{p(y)} \\ = \nabla_{x_t} \log p(x_t) + \nabla_{x_t} \log p(y|x_t) \quad (1)$$

Given that $p(y)$ is independent of x, it follows that $\nabla_{x_t} \log p(y) = 0$. To function effectively, this formulation requires an additional trained classifier model, $p(y|x_t)$. Following the approach in Song et al. [7], a score-based conditioning method is leveraged that connects diffusion models to denoised score-matching models [8]. Eq. 2 encapsulates this relation.

$$s_\theta(x_t, t, y) = \nabla_{x_t} \log p(x_t|y) = -\frac{1}{\sqrt{1-\bar{\alpha}_t}} \varepsilon_\theta(x_t, t, y) \quad (2)$$

By introducing a guidance scale ω, the influence of the classifier in the overall guidance process can be modulated, as expressed in Eq. 3.

$$s_\theta(x_t, t, y) = \nabla_{x_t} \log p(x_t) + \omega \nabla_{x_t} \log p(y|x_t) \quad (3)$$

Eq. 3 presents *Classifier Guidance models*; however, one major problem is that they need a separately trained classifier $p_\phi(y|x_t)$ that is capable of predicting the samples with varying degrees of noise x_t. To prevent this, the score function can be reformulated by replacing $\nabla_{x_t} \log p(y|x_t)$ in Eq. 3 with Eq. 1 to yield Eq. 4.

$$s_\theta(x_t, t, y) = (1-\omega)\nabla_{x_t} \log p(x_t) + \omega \nabla_{x_t} \log p(x_t|y) \quad (4)$$

This approach is called Classifier-Free Guidance (CFG). Leveraging the relation in Eq. 2, CFG can be expressed in terms of the noise diffusion model ε_θ (Eq. 5).

$$\tilde{\varepsilon}_\theta(x_t, t, y) = (1-\omega)\varepsilon_\theta(x_t, t) + \omega \varepsilon_\theta(x_t, t, y) \quad (5)$$

2) Sampling Process with CFG-DDIM: As mentioned in Sec. II-A1 of the main article, the CFG-DDIM is used to generate conditional guide wound images. The equation of this sampling process is shown in Eq. 6.

$$x_{t-1} = \sqrt{\bar{\alpha}_{t-1}} \left(\frac{x_t - \sqrt{1-\bar{\alpha}_t}\tilde{\varepsilon}_\theta(x_t, t, y)}{\sqrt{\bar{\alpha}_t}} \right) + \sqrt{1-\bar{\alpha}_{t-1}}\tilde{\varepsilon}_\theta(x_t, t, y) \quad (6)$$

Here, $\bar{\alpha}_t$ is the noise scaling factor at each step t. Algorithm 1 demonstrates the ConDiff Sampling process with CFG-DDIM.

Algorithm 1 ConDiff Sampling with CFG-DDIM

Require: Guide image: x_0, class label: y, guidance scale: ω, noise strength: t_0, and number of diffusion steps: T.

1: $x_{t_0 T} = x_0 + \sigma(t_0) z$, where $z \sim \mathcal{N}(0, I)$
2: **for** $t = t_0 T, \ldots, 1$ **do**
3: $\quad \tilde{\varepsilon}_\theta(x_t, t, y) = \omega \varepsilon_\theta(x_t, t, y) + (1-\omega)\varepsilon_\theta(x_t, t)$
4: $\quad x_{t-1} = \sqrt{\bar{\alpha}_{t-1}} \left(\frac{x_t - \sqrt{1-\bar{\alpha}_t}\tilde{\varepsilon}_\theta}{\sqrt{\bar{\alpha}_t}} \right) + \sqrt{1-\bar{\alpha}_{t-1}}\tilde{\varepsilon}_\theta$
5: **end for**
6: **return** $\hat{x}_0^{(y)}$

B. Implementation Details of the 2-Step Training Process

Training Stage 1 - Fine-Tuning the Diffusion Model: The diffusion model $\varepsilon_\theta(x_t, t, y)$ was fine-tuned using the MSE objective function. During each iteration, an image x_0 and its condition y were sampled from the wound training set D_r, with a diffusion step t sampled uniformly from $[1, \ldots, T]$ to create a noisy image x_t. The denoising U-Net ε_θ was trained for 10,000 iterations using the AdamW optimizer with a learning rate of 1×10^{-5}. After training, the ConDiff generator synthesized conditional DFU images with hyperparameters: guidance scale $\omega = 0.75$, noise strength $t_0 = 0.8$, and number of sampling steps $T = 30$. These synthetic images formed the dataset D_s, used in the second training stage.

Training Stage 2 - Training the Embedding Network f_ϕ: The embedding model f_ϕ, based on the EfficientNet-B0 architecture, was trained using both real dataset D_r and

synthetic dataset D_s. For each iteration, a batch of triplets $(x^{(a)}, x^{(p)}, x^{(n)})$ was sampled from D_r, with $(x^{(p)}, x^{(n)})$ being sampled from D_s with probability $p_{gen} = 0.2$. The model parameters ϕ were optimized to minimize the Triplet loss, effectively learning to distinguish between similar and dissimilar images (see Algorithm 2). The training involved 50 epochs with the AdamW optimizer, a learning rate of 1×10^{-3}, and the optimal parameters ϕ were selected based on the best validation performance.

Algorithm 2 Training a distance-based classifier model with Triplet loss

Require: embedding model: f_ϕ, real dataset: D_r, synthetic dataset: D_s, probability of sampling from D_s: p_{gen}, number of epochs: E, and batch size: B.
Ensure: learned f_ϕ

```
for epoch = 1,...,E do
    for batch = 1,...,⌈size(D_r)/B⌉ do
        (x^(a), x^(p), x^(n)) ~ D_r (sample B triplets)
        (x^(p), x^(n)) ~ D_s with probability p_gen
        Compute L_triplet.
        Take gradient step on ∇_φ L_triplet
    end for
end for
```

C. Evaluation Metrics

Model evaluations on the DFU infection dataset were analyzed as follows.

Classification metrics: The following metrics were used to assess the effectiveness of our proposed framework in the DFU infection classification task:

- **Accuracy** $ACC = \frac{TP+TN}{P+N}$, where TP is the number of true positive predictions, TN is the number of true negative predictions, P is the positive label (infected), and N is the negative label (not infected).
- **Sensitivity (SEN)** or recall reflects the proportion of actual positives that are correctly identified: $SEN = \frac{TP}{TP+FN}$, where FN denotes the number of false negative predictions.
- **Specificity (SPC)** reflects the proportion of actual negatives that are correctly identified: $SPC = \frac{TN}{TN+FP}$, where FP denotes the number of false positive predictions.
- **Positive Predictive Value (PPV)** or precision is the proportion of positive predictions that are true positives. $PPV = \frac{TP}{TP+FP}$.
- **F1-score** is the Harmonic Mean of Precision and Recall: $F1 = 2 \cdot \frac{PPV \cdot SEN}{PPV+SEN}$.

Image generation metrics: To evaluate the quality of synthetic images generated by each method, the Fréchet Inception Distance (FID) and the Inception Score (IS) were employed.

- **Fréchet Inception Distance (FID)** [9]: This metric assesses the quality of generated images by comparing them to real images. A lower FID score indicates greater similarity to real images, signifying higher quality and realism. The FID is calculated as:

$$FID = \|\mu - \mu_w\|^2 + \mathrm{tr}(\Sigma + \Sigma_w - 2(\Sigma\Sigma_w^{1/2}))$$

where $\mathcal{N}(\mu, \Sigma)$ is the multivariate normal distribution estimated from Inception-V3 features of real images, and $\mathcal{N}(\mu_w, \Sigma_w)$ is from generated images.
- **Inception Score (IS)** [10]: IS uses a pre-trained Inception v3 model to predict the class distribution of each generated image. Higher IS values indicate that the generated images are distinct and diverse. However, IS does not account for the distribution of real images, a known limitation. The IS is given by:

$$IS = \exp(\mathbb{E}_x[\mathrm{KL}(p(y|x)\|p(y))])$$

where $\mathrm{KL}(p(y|x)\|p(y))$ is the Kullback-Leibler divergence between the conditional distribution $p(y|x)$ and the marginal distribution $p(y)$.

D. Score-Weighted Class Activation Mapping (Score-CAM)

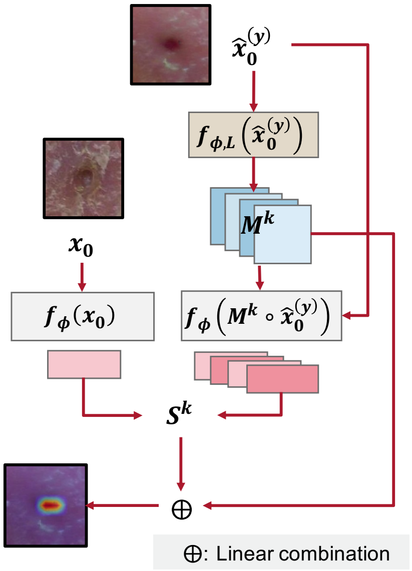

Fig. 1. Score-CAM pipeline for visualizing similarities between images

Score-CAM [11] is a method to interpret the decision-making process of CNN models in visual tasks. It creates a visual heatmap that reveals which regions of a conditional synthesized image $\hat{x}_0^{(y)}$ the embedding model f_ϕ found similar to a guide (input) image x_0. Score-CAM has the following steps: (1) extracting feature maps A^k from the last convolutional layer L; $A^k = f_{\phi,L}(\hat{x}_0^{(y)})$, (2) generating activation maps that emphasize predictive regions of the image; $M^k = \mathrm{Upsample}(A^k)$, (3) calculating cosine similarity scores between the guide image embedding and the corresponding synthesized images based on the activation maps; $S^k = \mathrm{Similarity}(f_\phi(x_0), f_\phi(M^k \circ \hat{x}_0^{(y)}))$, and (4) aggregating these activation maps into a comprehensive heatmap, with the weighting determined by the similarity scores $\alpha^k = \frac{\exp(S^k)}{\Sigma_j \exp(S_j^k)}$; $H_{\text{Score-CAM}} = \mathrm{ReLU}(\Sigma_k \alpha^k M^k)$. The visualization of the Score-CAM pipeline is shown in Fig. 1.

E. Ablation study and Image Generation Analysis

1) Effects of different sampling methods on infection classification: We compared the performance of the DFU infection

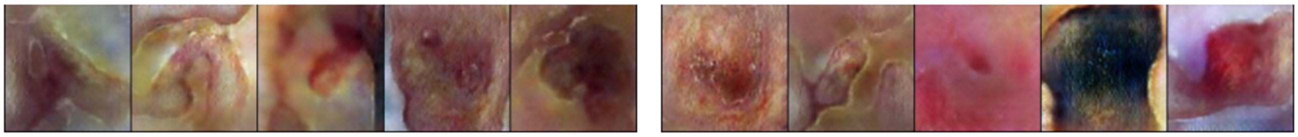

(a) cGAN generation: $FID = 11.201$, $IS = 2.966$

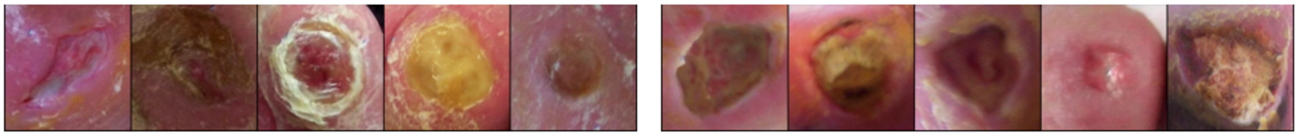

(b) ConDiff, DDIM sampling method: $FID = 2.917$, $IS = 3.662$

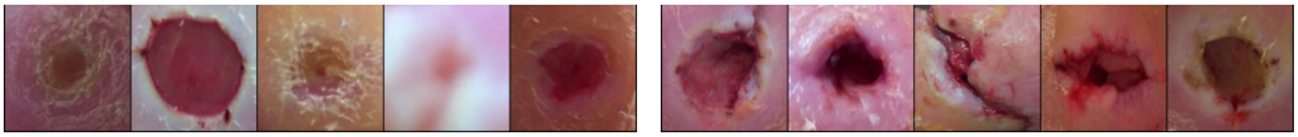

(c) ConDiff, CFG-DDIM sampling method with $\omega = 3$: $FID = 3.779$, $IS = 3.669$

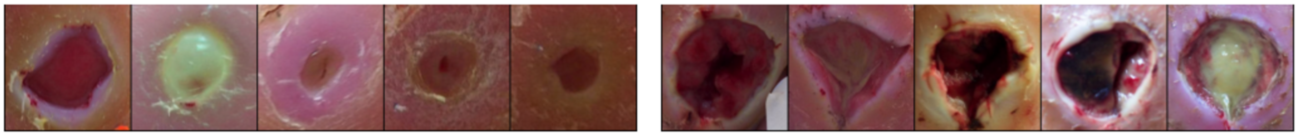

(d) ConDiff, CFG-DDIM sampling method with $\omega = 7.5$: $FID = 5.068$, $IS = 3.885$

Fig. 2. Guided Image Generation. Condition on the label: (Left) uninfected wound and (Right) infected wound.

classification when two different sampling approaches were applied to ConDiff; (1) DDIM sampling and (2) CFG-DDIM sampling. Note that the difference between these 2 approaches is that the $\tilde{\varepsilon}_\theta(x_t, t, y)$ (in Line.3 of Algorithm 1) for the DIIM process is just $\varepsilon_\theta(x_t, t, y)$ as it does not involve a guidance scale ω while the CFG-DDIM process does include ω.

Table. I shows that the use of CFG-DDIM sampling significantly outperforms DDIM sampling in infection classification. This is because the guided generated images between 2 labels by DDIM are not different enough to make D_ϕ (Eq. 5 of the main article) determine which of the synthesized images is most similar to the input image. The visualization and quality of synthesized images are shown in the Supplementary Material.

TABLE I. Comparison of DFU infection classification by ConDiff Classifier with various sampling methods.

Method	Acc	F1	SEN	SPEC	PPV
DDIM	0.603	0.635	0.581	0.641	0.699
CFG-DDIM	**0.833**	**0.858**	**0.858**	**0.796**	**0.858**

2) Evaluation of Conditional Image Synthesis: In Sec -E1, it was established that CFG-DDIM sampling is more effective for distance-based classification. However, realistic DFU images can still be generated using the DDIM sampling method.

Table II reveals that image synthesis using ConDiff + DDIM results in the lowest FID score, indicating that the distribution of the images synthesized closely resembles that of the real data. However, the Inception Score (IS) for the ConDiff + DDIM approach is lower than the ConDiff + CFG-DDIM approach. This finding aligns with Ho et al.'s experiment [3], highlighting a trade-off between FID and IS. In our context, the IS reflects the ease of differentiating between conditional synthesized images. Consequently, the ConDiff Classifier using the DDIM sampling approach underperforms relative to the CFG-DDIM sampling approach, as seen in Table I. This is attributed to the challenge in label clarification due to the lower IS. Note that IS is not significantly different across our diffusion models because the IS metric considers the clarity and diversity of synthesized images, as shown in Fig. 2. Additionally, the experiment employed the popular conditional GAN approach [12] for generating DFU images conditioned on infection status, providing a comparison to diffusion methods. The results indicate that the quality of synthetic images using conditional GAN is inferior to those produced by diffusion methods.

TABLE II. Quality measurement of conditional synthesized images by generative models.

Model	FID Score ↓	IS ↑
ConDiff + DDIM	**2.917**	3.662
ConDiff + CFG-DDIM, $\omega = 3$	3.779	3.669
ConDiff + CFG-DDIM, $\omega = 7.5$	5.068	**3.885**
Conditional GAN [12]	11.201	2.965

References

[1] R. Rombach, A. Blattmann, D. Lorenz, P. Esser, and B. Ommer, "High-resolution image synthesis with latent diffusion models," in *Proceedings of the IEEE/CVF conference on computer vision and pattern recognition*, 2022, pp. 10 684–10 695.

[2] P. Dhariwal and A. Nichol, "Diffusion models beat gans on image synthesis," *Advances in neural information processing systems*, vol. 34, pp. 8780–8794, 2021.

[3] J. Ho and T. Salimans, "Classifier-free diffusion guidance," *arXiv preprint arXiv:2207.12598*, 2022.

[4] W. Peebles and S. Xie, "Scalable diffusion models with transformers," in *Proceedings of the IEEE/CVF International Conference on Computer Vision*, 2023, pp. 4195–4205.

[5] A. Nichol, P. Dhariwal, A. Ramesh, P. Shyam, P. Mishkin, B. McGrew, I. Sutskever, and M. Chen, "Glide: Towards photorealistic image generation and editing with text-guided diffusion models," *arXiv preprint arXiv:2112.10741*, 2021.

[6] C. M. Bishop and H. Bishop, "Deep neural networks," in *Deep Learning: Foundations and Concepts*. Springer, 2023, pp. 171–207.

[7] Y. Song and S. Ermon, "Generative modeling by estimating gradients of the data distribution," *Advances in neural information processing systems*, vol. 32, 2019.

[8] Y. Song, J. Sohl-Dickstein, D. P. Kingma, A. Kumar, S. Ermon, and B. Poole, "Score-based generative modeling through stochastic differential equations," *arXiv preprint arXiv:2011.13456*, 2020.

[9] M. Heusel, H. Ramsauer, T. Unterthiner, B. Nessler, and S. Hochreiter, "Gans trained by a two time-scale update rule converge to a local nash equilibrium," *Advances in neural information processing systems*, vol. 30, 2017.

[10] T. Salimans, I. Goodfellow, W. Zaremba, V. Cheung, A. Radford, and X. Chen, "Improved techniques for training gans," *Advances in neural information processing systems*, vol. 29, 2016.

[11] H. Wang, Z. Wang, M. Du, F. Yang, Z. Zhang, S. Ding, P. Mardziel, and X. Hu, "Score-cam: Score-weighted visual explanations for convolutional neural networks," in *Proceedings of the IEEE/CVF conference on computer vision and pattern recognition workshops*, 2020, pp. 24–25.

[12] M. Mirza and S. Osindero, "Conditional generative adversarial nets," *arXiv preprint arXiv:1411.1784*, 2014.
